# Brazilian green propolis modulates inflammation, angiogenesis and fibrogenesis in intraperitoneal implant in mice

**DOI:** 10.1186/1472-6882-14-177

**Published:** 2014-05-29

**Authors:** Luiza DC Lima, Silvia P Andrade, Paula P Campos, Lucíola S Barcelos, Frederico M Soriani, Sandra AL Moura, Mônica AND Ferreira

**Affiliations:** 1 Department of Physiology and Biophysics, Institute of Biological Sciences, Federal University of Minas Gerais (UFMG), Antônio Carlos 6627- Pampulha, Belo Horizonte, Minas Gerais CEP 31.270-901, Brazil; 2 Department General Pathology, Institute of Biological Sciences, Federal University of Minas Gerais (UFMG), Antônio Carlos 6627- Pampulha, Belo Horizonte, Minas Gerais CEP 31.270-901, Brazil; 3 Center of Research in Biological Science, Federal University of Ouro Preto (UFOP), Ouro Preto, Minas Gerais, Brazil

**Keywords:** Water extract propolis, Cytokines, Macrophage activation, Fibrosis

## Abstract

**Background:**

Chronic inflammatory processes in the peritoneal cavity develop as a result of ischemia, foreign body reaction, and trauma. Brazilian green propolis, a beeswax product, has been shown to exhibit multiple actions on inflammation and tissue repair. Our aim was to investigate the effects of this natural product on the inflammatory, angiogenic, and fibrogenic components of the peritoneal fibroproliferative tissue induced by a synthetic matrix.

**Methods:**

Chronic inflammation was induced by placing polyether-polyurethane sponge discs in the abdominal cavity of anesthetized Swiss mice. Oral administration of propolis (500/mg/kg/day) by gavage started 24 hours after injury for four days. The effect of propolis on peritoneal permeability was evaluated through fluorescein diffusion rate 4 days post implantation. The effects of propolis on the inflammatory (myeloperoxidase and n-acetyl-β-D-glucosaminidase activities and TNF-α levels), angiogenic (hemoglobin content-Hb), and fibrogenic (TGF-β1 and collagen deposition) components of the fibrovascular tissue in the implants were determined 5 days after the injury.

**Results:**

Propolis was able to decrease intraperitoneal permeability. The time taken for fluorescence to peak in the systemic circulation was 20 ± 1 min in the treated group in contrast with 15 ± 1 min in the control group. In addition, the treatment was shown to down-regulate angiogenesis (Hb content) and fibrosis by decreasing TGF-β1 levels and collagen deposition in fibroproliferative tissue induced by the synthetic implants. Conversely, the treatment up-regulated inflammatory enzyme activities, TNF-α levels and gene expression of NOS2 and IFN-γ (23 and 7 fold, respectively), and of FIZZ1 and YM1 (8 and 2 fold) when compared with the untreated group.

**Conclusions:**

These observations show for the first time the effects of propolis modulating intraperitoneal inflammatory angiogenesis in mice and disclose important action mechanisms of the compound (downregulation of angiogenic components and activation of murine macrophage pathways).

## Background

Chronic inflammatory processes in the peritoneal cavity are reported to develop as a result of ischemia, foreign body reaction, and trauma to the peritoneum. Platelet accumulation and activation at lesion sites are the first events to take place followed by coagulum formation that provides a framework for inflammatory cells attachment, angiogenesis, and fibroblast proliferation. If the stimulus remains, persistent activation of inflammatory and angiogenic cascades occurs [1-3]. It has been proposed that attenuation and/or inhibition of one or more components of the processes associated with this type of injury would represent key targets for controlling chronic inflammation in the peritoneal cavity. Therefore, the search for compounds with potential anti-inflammatory, anti-angiogenic, and anti-fibrogenic properties has gained clinical importance [3,4].

Several groups have reported that local or systemic administration of propolis extract exerts immunomodulatory, antimicrobial, anti-inflammatory, antioxidant, and antiangiogenic effects on a number of pathological conditions, including accelerating repair phases in various experimental wound healing models. Furthermore, this natural beehive product presents minimal adverse effects and no toxicity [5-8]. Ethanol extract of propolis (EEP) is most commonly studied in propolis research. In contrast, water extract of propolis (WEP) has rarely been investigated, even though WEP and its main compounds (including caffeoylquinic acids) have greater antioxidant effects, inhibitory activity against some enzymes, and absorbency than do EEP and its compounds (15).

We have recently reported that implantation of a synthetic matrix in the peritoneal cavity in mice induced a fibroproliferative process whose components, inflammatory cell recruitment, angiogenesis, and extracellular matrix deposition were identified and modulated by various compounds [9,10]. We hypothesized that propolis might be of potential therapeutic value in modulating the components of these lesions in our model. In addition, considering that cytokines/chemokines play critical roles in fibroproliferative processes [11-13] and peritoneal macrophages express traits associated with both classical and alternative activation gene expression [14,15], we set out to study a possible effect of propolis in modulating these parameters in sponge-induced intraperitoneal inflammatory angiogenesis.

## Methods

### Animals

Male Swiss mice 7-8 weeks (20-30 g body weight) provided by the Central Animal Facility at the ICB, UFMG were used in these experiments. The animals were housed individually and provided with chow pellets and water *ad libitum*. The light/dark cycle was 12:12 h with lights on at 7:00 a.m. and lights off at 7:00 pm. Efforts were made to avoid all unnecessary distress to the animals. Housing, anesthesia and postoperative care concurred with the guidelines established by our local Institutional Animal Welfare Committee.

All experiments were performed according to the Ethical Guidelines for Experimental Animal Investigation and approved by the Ethics Committee on Animal Experimentation of the Federal University of Minas Gerais (CETEA/UFMG), protocol number 248/08.

### Preparation of the aqueous extract of green propolis

Propolis samples were collected from September 2005 to September 2006 in the municipality of Jaguaraçu, Minas Gerais, Brazil from *Apis mellifera* hives. Samples were homogenized and frozen at –18°C and an aliquot (200 g) was powdered and 500 ml of distilled water added. The suspension was maintained for 30–60 min while stirring at 70°C and then cooled at room temperature. The supernatant was filtered through Whatman 1 filter paper to obtain the first extract. The residue was treated again using the same procedure, from which a second extract was obtained. The two extracts were pooled and then lyophilized. Analysis of the chemical composition of propolis samples have been published elsewhere [16].

### Preparation of sponge discs, implantation, and treatment

Polyether-polyurethane sponge (Vitafoam Ltd., Manchester, UK) was used as the implanted material. The implants were in the shape of discs, 5 mm thick × 8 mm diameter, and soaked overnight in 70% v/v ethanol and then sterilized by boiling in distilled water for 15 minutes before implantation. The animals were anesthetized by intraperitoneal injection of 4 μL/g of a mixture of ketamine (150 mg/kg) and xylazine (10 mg/kg) and the ventral hair shaved and the skin wiped with 70% ethanol. The sponge discs were aseptically placed in the peritoneal cavity by means of a 1 cm long mid-line incision in the linea alba of the abdomen. Post-operatively, the animals were monitored for any signs of infection at the surgical site, discomfort or distress. Brazilian green propolis (Baccharis dracunculifolia DC (Asteraceae) found in Jaguaraçu, Minas Gerais , Brazil; was processed by crushing 200 g of the resinous compound and adding 500 ml of distilled water to yield green propolis extract (WEP). The main constituents of the aqueous extract as determined by HPLC/MS/MS are mono- and di-O-caffeoylquinic acids as previously described [16]. The treated groups received WEP (500 mg/kg/day) orally by gavage. Treatment started immediately after the injury and lasted for 4 days. The control group received water in the same schedule. We used a total of 73 mice for the entire study. Compound dosage was chosen based on pilot experiments and data from the literature [16]. Treatment was well tolerated by the mice during the experimental period.

### Assessment of intraperitoneal permeability in Swiss mice

We reasoned that the surgical implantation of a sponge disc in the intraperitoneal cavity would alter permeability locally and that propolis treatment would modulate this response. For this series of experiments, the assay was performed in anesthetized mice at day 4 post-implantation after the fourth dose of propolis. We used the sodium fluorescein outflow rate to assess local blood flow [17]. A sterile solution (10 μL) of sodium fluorescein (Sigma, USA; 1%) was injected intraperitoneally. Blood samples (5 μL) were withdrawn from the tail vein at 1, 3, 5, 7, 10, 15, 20, 25 and 30 min after dye injection. Blood samples were mixed in 1 mL of isotonic saline, centrifuged for 5 min, and the supernatant was kept for fluorescence determination in a Jenway fluorimeter (model 6200) at an excitation/emission of 485/520. The results were expressed as the peak of the fluorescence signal in the systemic circulation (min). Five animals from each group (control and propolis) were used to establish this parameter.

### Hemoglobin extraction

The extent of vascularization of the sponge implants was assessed by the amount of hemoglobin detected in the tissue using the Drabkin method [9,18]. Five days post-implantation, the animals (n = 8-10 per group) were killed and the sponge implants and surrounding fibrous tissue were carefully removed, dissected free from adherent tissue, weighed homogenized (Tekmar TR-10, OH) in 5 mL of Drabkin reagent (Labtest, São Paulo, Brazil) and centrifuged at 12000 × *g* for 20 min. The supernatants were filtered through a 0.22-μm Millipore filter. The hemoglobin concentration in the samples was determined spectrophotometrically by measuring absorbance at 540 nm using an ELISA plate reader and compared against a standard hemoglobin curve. Hemoglobin content in the implant was expressed as μg Hb per mg wet tissue.

### Tissue extraction and determination of myeloperoxidase and N-acetyl-β-D-glucosaminidase activities

The extent of neutrophil accumulation in the implants was measured by assaying myeloperoxidase (MPO) activity as previously described [9-20]. This enzyme is present at a high specific activity within neutrophils [20]. After processing the supernatant of the implants for hemoglobin determination (see above), a part of the corresponding pellet was weighed, homogenized in pH 4.7 buffer (0.1 M NaCl, 0.02 M Na_3_PO_4_, 0.015 M Na_2_EDTA), and centrifuged at 12,000 × *g* for 10 min. The pellets were then re-suspended in a 0.05 M sodium phosphate buffer (pH 5.4) containing 0.5% hexa-decyltrimethylammonium bromide (HTAB) followed by three freeze-thaw cycles using liquid nitrogen. MPO activity in the supernatant samples was assayed by measuring the change in absorbance (optical density; OD) at 450 nm using tetramethylbenzidine (1.6 mM) diluted in DMSO (dimethyl sulfoxide) and H_2_O_2_ (0.3 mM). The reaction was terminated by adding 50 μL of H_2_SO_4_ (4 M). Results were expressed as a change in OD per mg of wet tissue (implant).

Infiltration of mononuclear cells was quantitated by measuring the levels of the lysosomal enzyme N-acetyl-β-D-glucosaminidase (NAG) present in high levels in activated macrophages [9-20]. Part of the pellet remaining after hemoglobin measurement was kept for this assay. These pellets were weighed, homogenized in NaCl solution (0.9% w/v) containing 0.1% v/v Triton X-100 (Promega; USA) and centrifuged (3,000 × *g*; 10 min at 4°C). Samples of the resulting supernatant (100 μL) were incubated for 30 min with 100 μL of p-nitrophenyl-N-acetyl-beta-D-glucosaminide (Sigma; USA) prepared in a citrate/sodium phosphate buffer (0.1 M citric acid, 0.1 M Na_2_HPO_4_; pH4.5) to yield a final concentration of 2.24 mM. The reaction was stopped by adding 100 μl of a glycine buffer (0.8 M glycine, 0.8 M NaCl, 0.8 M NaOH; pH 10.6). Hydrolysis of the substrate was determined by measuring the absorption at 400 nm. NAG activity was expressed as nmol of p-nitrophenol per mg wet tissue.

### Collagen measurement

Total soluble collagen was measured in whole tissue homogenates by the Sirius Red reagent based-assay [9,21]. The tissue from 5 animals per group was homogenized in 1 mL of PBS and 50 μL of the sample was mixed with 50 μL of Sirius Red reagent. The collagen-dye complex was precipitated by centrifugation at 5,000 × *g* for 10 min. The supernatants were drained off, discarded, and the pellet washed with 500 μL of ethanol (99% pure and methanol free). One mL of a 0.5 M NaOH solution was added to the remaining collagen-bound dye pellet. Color intensity of the samples was measured at 540 nm. The calibration curve was set up on the basis of the gelatin standard (Merck). Results are expressed as μg collagen/mg wet tissue.

### Measurement of VEGF, TNF-α and TGF-β1 production

For this procedure, 10 animals in each group were used. The implants were removed at day 5 post implantation, homogenized in PBS pH 7.4 containing 0.05% Tween-20 (Difco/USA), and centrifuged at 4°C, 10,000 × *g* for 30 min. The cytokines VEGF, TNF-α and TGF-β1 in the supernatant from each tissue were measured in 50 μL of the supernatant using Immunoassay Kits (R & D Systems, USA) and following the manufacturer’s protocol [9].

### Histological analysis and staining

The sponge implants from both groups (control and propolis-treated, n = 3 in each group) were carefully excised, dissected free of adherent tissue, fixed in 10% buffered formalin (pH 7.4) and processed for paraffin embedding. Sections with a thickness of 5 mm were stained with Gomori Trichrome for light microscopic studies. This staining is used to visualize collagen fibers (reddish) and blood vessel-like structures.

### Real time PCR analysis

Implants from mice of propolis-treated (n = 5) and non-treated groups (n = 4) at day 5 postimplantation were analyzed to determine the expression of genes associated with innate and alternative macrophage activation macrophage activation [22]. Total RNA was obtained using Trizol (Invitrogen, Carlsbad, CA, USA) according to the procedure supplied by the manufacturer. Total RNA was reverse transcribed with SuperScript III (Invitrogen) as described by the manufacturer. Real-time quantitative PCR was performed on an ABI PRISM Step-One sequence-detection system (Applied Biosystems, Carlsbad, CA) using SYBR Green PCR Master Mix (Applied Biosystems). The relative expression level of genes was determined by the 2(-delta delta Ct) method and data were normalized by 18S ribosome subunit expression levels. All reactions were replicated. Primers were generated for nos2 (forward: AGC ACT TTG GGT GAC CAC CAG GA, reverse: AGC TAA GTA TTA GAG CGG CGG CA), ifn- (forward: ACA ATG AAC GCT ACA CAC TGC AT, reverse: TGG CAG TAA CAG CCA GAA ACA), fizz1 (forward: ACC TTT CCT GAG ATT CTG CCC, reverse: CAG TGG TCC AGT CAA CGA GTA AGC), ym1 (forward: GGC TAC ACT GGA GAA AAT AGT CCC, reverse: CCA ACC CAC TCA TTA CCC TGA TAG) and 18S (forward: CGT TCC ACC AAC TAA GAA CG, reverse: CTC AAC ACG GGA AAC CTC AC).

### Statistical analysis

All the data were expressed as mean ± SEM. All data analyzed were submitted to the Kolmogorov-Smirnov test and the variables presented normal distribution. Comparison between groups was made by performing a T-test using GraphPad Prism version 4.00 for Windows (GraphPad Software, San Diego California USA, http://www.graphpad.com). Differences between means were considered significant when p value was < 0.05.

## Results

### Chemical analyses

The main constituents of the aqueous extract of the analyzed propolis sample are caffeoylquinic acids: 4,5-di-O-caffeoylquinic (27.2%); 3,4-di-O-(E)-caffeoylquinic (15.8%); didihydrocaffeoylquinic (13.8%) and 3,5-di-O-(E)-caffeoylquinic (8.0%). Other caffeoylquinic acids, such as 4-O-(E)-caffeoylquinic acid, 5-O-(E)-caffeoylquinic acid, and cinnamic acid derivatives, such as artepillin C and drupanin, are minor constituents (2.6, 4.4, 5.0, and 2.0%, respectively) [16].

**Figure 1:**
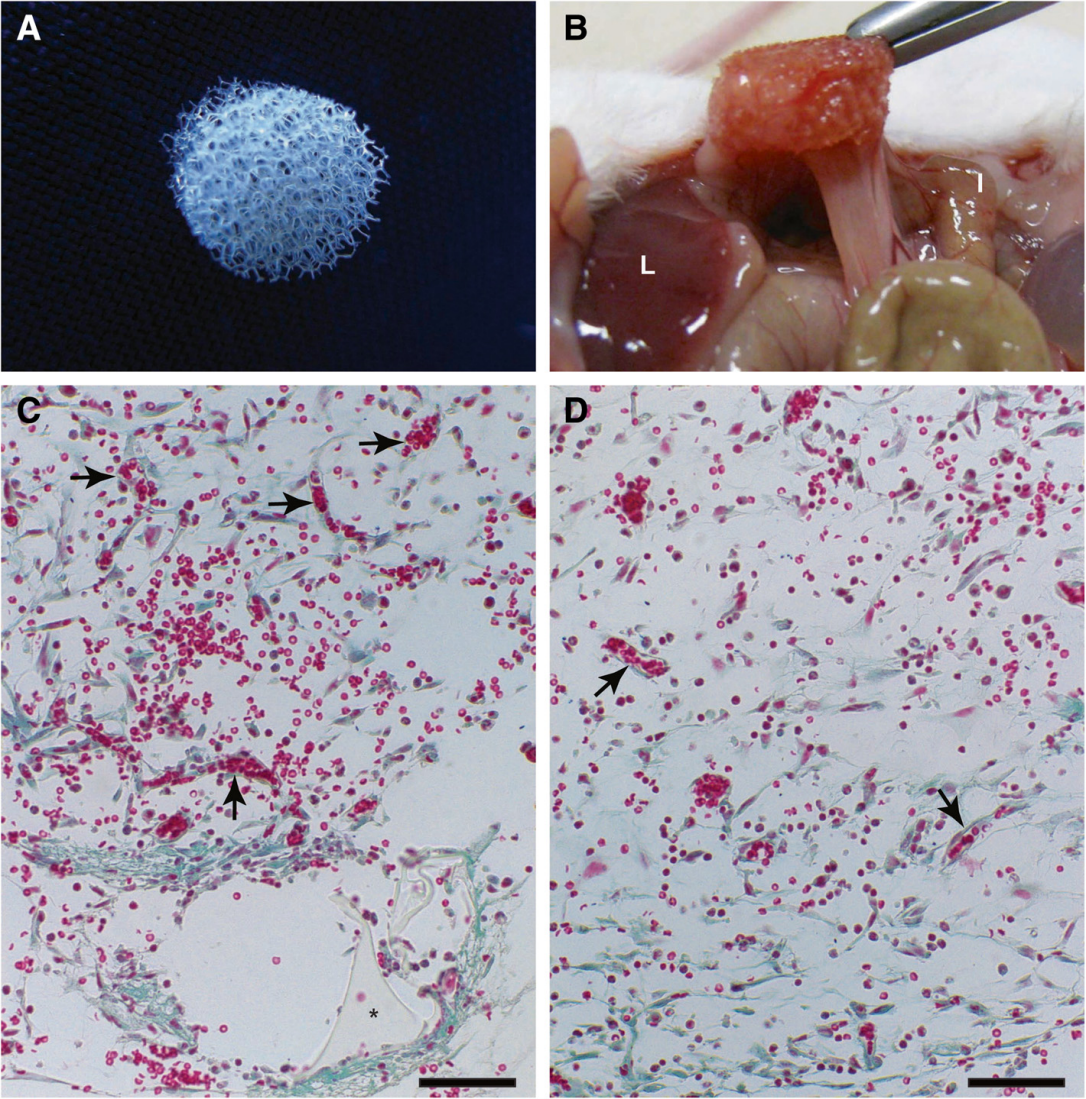
**Representative images of the sponge implant disc.** Sponge disc before implantation in the peritoneal cavity **(A)**. In **(B)** sponge disc 5 days after implantation. The implant is extensively adhered to the intestine and liver by a bridge of fibrous tissue. In **C** and **D** representative histological sections (5 μm, stained with Gomori Trichrome) of intraperitoneal implant. The matrix of the synthetic sponge is occupied with inflammatory cells, spindle-shaped cells and blood vessels. The fibrovascular tissue in implants of non-treated mice **(C)** is denser and more vascularized than the treated implant **(D)**. Black arrow: blood vessels; *: The sponge material is seen as triangular objects under the microscope; 60x; Bar: 50 μm.

Systemic administration of propolis (500 mg/kg/200 μL) for 4 days showed no signs of toxicity such as weight loss, sedation, or changes in the animals’ motor activity. The surgical procedure and sponge matrix implantation were well tolerated by all animals, causing no infection or rejection yet inducing adhesion-like tissue.The intraperitoneal implants were enveloped by a fibrous capsule and firmly adherent to visceral organs (liver and/or intestines) by day 5. Figures 1A and B show the aspect of the sponge discs before and after implantation (5 days). Histological analysis (H&E) showed that this procedure induced a fibrovascular response causing the synthetic sponge matrix to be filled with newly-formed tissue. The intraperitoneal implants were infiltrated by fibrovascular stroma occupying the entire sponge by day 5. The tissue was composed of an inflammatory infiltrate with various cell types such as leukocytes, mesothelial-like cells, and microvessels (Figure 1C). In the propolis-treated group, vascularization of the implants was decreased and inflammatory cell infiltrate showed no visible histological change when compared with the implants from non-treated animals (Figure 1D).

### Effects of propolis on diffusion rate of sodium fluorescein applied intraperitoneally

**Figure 2:**
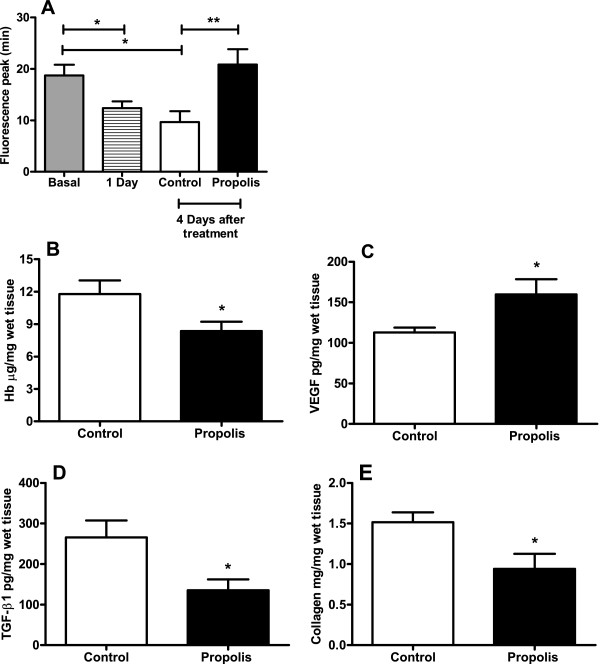
**Effects of WEP on diffusion rate.** WEP (500 mg/kg/day) on diffusion rate of sodium fluorescein applied intraperitoneally; **(A)**, on angiogenesis (**B** and **C**) and on fibrogenesis components of intraperitoneal implants (**D** and **E**). Values shown are the means (±SEM) from f 10 animals in both groups. *p < 0.05 versus control group.

Systemic treatment with propolis for 4 days was able to lower the rate of fluorescein diffusion from the peritoneal cavity to the systemic circulation when compared with the untreated animals. It took 20 ± 1 min for the fluorescence to peak in the systemic circulation in the treated group and 15 ± 1 min in the control group (Figure 2A).

### Measurement of anti-angiogenic effects

Treatment with propolis (500 mg/kg/200 μL) reduced implant neovascularization, as detected by changes in the hemoglobin content. A decrease in hemoglobin (control 11.8 ± 1.3 versus propolis 8.4 ± 0.9 μg/mg wet tissue) was observed after treatment (29%) (Figure 2B). VEGF levels intraimplant did not follow HB reduction; instead, VEGF production increased after propolis treatment (control 112.8 ± 5.9 versus propolis 159.7 ± 18.7 pg/mg wet tissue) (Figure 2C).

### Measurement of fibrogenic parameters

The levels of TGF-β1 (control 265.9 ± 41.6 versus propolis 135.6 ± 26.7 pg/mg wet tissue) and collagen content (control 1.5 ± 0.1 versus propolis 0.9 ± 0.2 μg/ mg wet tissue) as assessed by picrossirius colorimetric assay decreased in the propolis-treated group, in contrast with the control group (Figure 2D and E).

### Effect of propolis on inflammatory parameters of intraperitoneal implants

**Figure 3:**
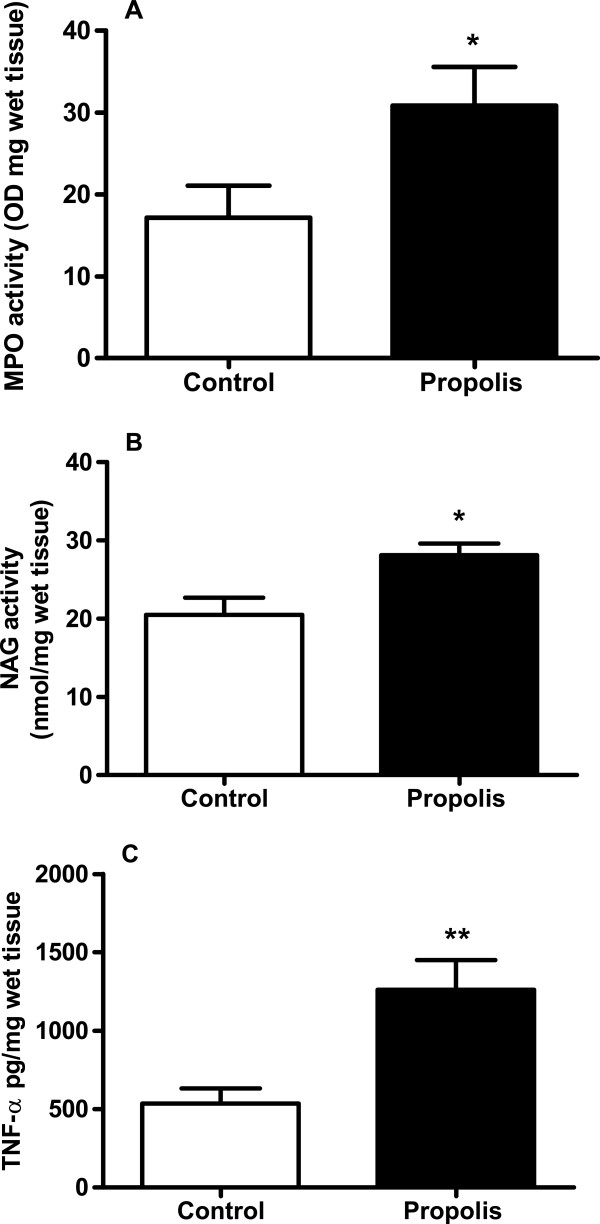
**Effects of WEP (500 mg/kg/day) on the inflammatory component of intraperitoneal implant.** Neutrophil accumulation **(A)**, macrophage accumulation **(B)** and TNF-α levels **(C)**. Values shown are means (±SEM) from 8-10 animals in each group. *p < 0.05 versus control group.

Inflammatory components of the intraperitoneal implants were determined by estimating the numbers of leukocytes in the implants. Neutrophil numbers (as MPO activity - control 17.2 ± 3.9 versus propolis 30.8 ± 4.8 OD/mg wet tissue), macrophage accumulation (as NAG activity - control 20.5 ± 2.2 versus propolis 28.1 ± 1.5 nmol/mg wet tissue), and TNF-α (control 536.0 ± 95.7 versus propolis 1263 ± 188.6 pg/ mg wet tissue) levels increased by about 50%, 10%, and 60%, respectively, after propolis treatment (Figure 3A-C).

### Effect of propolis on macrophage-associated genes in intraperitoneal implants

**Figure 4:**
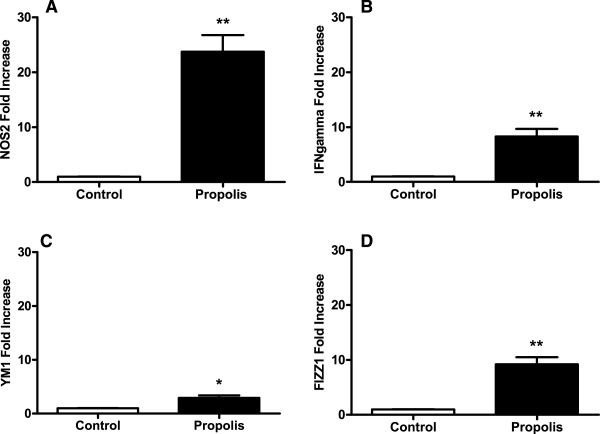
**Effects of WEP (500 mg/kg/day) on macrophage-associated cytokines/genes in intraperitoneal implant.** mRNA expression of *nos2***(A)**, *ifn-***(B)**, *ym1***(C)**, and *fizz1***(D)**. The treatment up-regulates classical and alternative macrophage activation-associated genes in relation to the control animals. Values are the means (±SEM) from groups of 4-5 animals. ** P < 0.01; *P < 0.05 versus control group.

As implant macrophage content was modulated by propolis, we analyzed the expression of genes associated with macrophage differential activation. The alternative macrophage activation-associated markers *FIZZ1* and *YM1*, as well as the expression of the classical macrophage activation-associated genes *NOS2* or *IFN-*γ, were significantly up-regulated in the propolis-treated group when compared with the control group (Figure 4A-D). Although an increase in both pathways was observed, the classical activated macrophages pathway was much higher (23-fold) for NOS2. A 7-fold increase was obtained for IFN-γ expression. In the alternative macrophage pathway, the increase in FIZZ1 expression was higher (8-fold) than in YM1 (2-fold).

## Discussion

We have previously described a model of chronic intraperitoneal inflammation based on a polyether-polyurethane sponge matrix implantation technique. The intraperitoneal implants were shown to be firmly adhered to surrounding organs (liver and/or intestines), highly vascularized, infiltrated with inflammatory cells, and producing pro-inflammatory and pro-angiogenic cytokines [9,10].

Using this model, the data presented here demonstrate, for the first time, that the systemic treatment with water extract of propolis (WEP) reduced vascular permeability, angiogenesis, and fibrosis of intraperitoneal implants. Histological analysis of the tissue corroborated our biochemical findings indicating the anti-angiogenic and antifibrogenic effects of propolis. Conversely, propolis was shown to increase all the inflammatory parameters analyzed, neutrophil and macrophage recruitment, TNF-α production, and gene expression of classical and alternative macrophage activation. Several groups have reported that local or systemic administration of propolis extracts exert immunomodulatory, antimicrobial, anti-inflammatory, antioxidant, antiangiogenic, and pro-healing activity in various experimental models [5-8].

At least in part, this discrepancy may be attributed to a number of factors, such as animal models, type of injury, manner of administration, dosage, duration of the experiments and of the source and type of propolis sample. Indeed, variations in propolis formulation have been reported, related to the plants in the geographic region from which propolis has been collected [16]. To some extent, our results are in agreement with the effects of propolis in other experimental models, for example its anti-angiogenic action. Interestingly, VEGF production was augmented in the propolis-treated group. This increase in pro-angiogenic cytokine may have been an attempt to compensate for decreased blood flow in the injury. It is intriguing that the reported anti-inflammatory effects of propolis are in contrast with the results presented here, particularly those from our group, which showed propolis to exert an anti-inflammatory effect on implants located subcutaneously in mice [16]. We have previously demonstrated that the anatomical site markedly influences the host response to a synthetic matrix [19] and, herein, we extend this observation to the host response to a pharmacological compound. It is striking that by up-regulating pro-inflammatory pathways, propolis has down-regulated angiogenesis and fibrosis in the peritoneal implant. To some extent, our results challenge the notion of the co-dependence of angiogenesis and inflammation in the maintenance of fibroproliferative pathological processes [23]. It may be that the co-dependence exists in many, but not in all, pathological processes, especially those in the peritoneal cavity. In this context, it is worth pointing out that the factors involved in healing peritoneal injuries are dissimilar to healing in other anatomical compartments. For instance, mesothelial cells are the specific cell type activated after injury to the peritoneum and responsible for the release of inflammatory mediators, chemokines, and cytokines that, in turn, recruit inflammatory cells. Activated mesothelial cells produce excessive plasminogen activator inhibitor-2 when compared with activated endothelial cells [24]. Macrophages from the peritoneal cavity display a range of features that differs from macrophages in other sites [14]. Likewise, fibroblasts in human tissue from the peritoneal cavity develop a specific phenotype expressing cyclooxygenase-2 [25]. Furthermore, while many phases of wound repair and mechanisms that regulate this process are common to many types of wounds, there are differences between dermal and peritoneal healing. Dermal injuries heal inward from the edges and the rate of healing depends on the size of the lesion, whereas peritoneal wounds heal simultaneously throughout the lesion, and the rate is independent of the injury’s surface [26]. Thus, it seems pertinent to attribute the different responses to the same compound to all these inherent dissimilarities between the cells and healing processes in injuries in different anatomical sites.

Another finding that has emerged from our study was the effect of propolis on macrophage activation states. Generically, these states are classified as inflammatory/classical (stimulated by TNF-α and IFN-γ) or alternative (expressing YM1 and FIZZ1), depending on a variety of environmental factors (cytokines, pattern recognition receptors, hormones) [14,15]. Interestingly, propolis up-regulated both classical and alternative pathways, yielding approximately 23- and 7-fold increases in the NOS2/IFN-γ and 8- and 2-fold increases in the FIZZ1/YM1 cytokine/gene expression. By simultaneously activating the inflammatory and alternative macrophage states, propolis treatment decreased neovascularization and fibrosis (key components in the maintenance of chronic fibroproliferative processes) while, at the same time, increasing the pro-inflammatory markers and cytokine (TNF-α) involved in fibrinolytic activity. This phenotypic heterogeneity in macrophage responses to this compound is fully compatible with the opposing functions of this cell in repair processes (proinflammatory versus anti-inflammatory, tissue-repair versus tissue-destruction) [14,27]. Therefore, it is likely that propolis exerted selective actions amplifying inflammatory signals that led to the increased production of “benign” pro-inflammatory molecules by the various cell types in the implant microenvironment. Conversely, the down-regulation of pro-fibrogenic signals (TGF-β1 production) resulted in decreased collagen deposition in the implant compartment. Negative modulation of these molecules is fully compatible with the notion that a decrease in both components results in fibrosis resolution [28]. The net effect of propolis treatment was the attenuation of angiogenesis and fibrosis in intraperitoneal implant.

## Conclusions

The main constituents of the aqueous extract of green propolis from Southeast Brazil were mono- and di-O-caffeoylquinic acids and phenylpropanoids [16]. It is tempting to speculate that these components were the active factors that modulated the main cell types involved in the effects of the compound. Interestingly, the active component (s) in the WEP sample responsible for modulating angiogenesis and inflammation in intraperitoneal fibrovascular tissue seemed to exert specific actions depending on the injury site. The observations described here show the effects of propolis modulating implant-induced intraperitoneal inflammatory angiogenesis in mice for the first time and reveal important mechanisms of the compound (down-regulation of angiogenic components and activation of murine macrophage pathways).

## Abbreviations

MPO: Myeloperoxidase; NAG: N-acetyl-β-D-glucosaminidase; TGF-β1: Transforming growth factor beta 1; TNF-α: Tumor necrosis factor-alpha; VEGF: Vascular endothelial growth factor; NOS2: Nitric oxide synthase 2; IFN- or ifn-: Interferon gamma; WEP: Extract of green propolis; HTAB: Hexa-decyltrimethylammonium bromide; DMSO: Dimethyl sulfoxide; OD: Optical density; TMB: Tetramethylbenzidine; H&E: Hematoxylin and eosin stain; Hb: Hemoglobin; IL: Interleukin; PCR: Polymerasechain reaction.

## Competing interest

The authors declare that they have no competing interests.

## Authors’ contributions

All authors participated in the design, interpretation of the results and analysis of the data, and review of the manuscript: LDCL, PPC, FMS, SALM conducted the experiments and LSB, MANDF, and SPA wrote the manuscript. All authors read and approved the final manuscript.

## Pre-publication history

The pre-publication history for this paper can be accessed here:

http://www.biomedcentral.com/1472-6882/14/177/prepub
